# New Insights into the Chemical and Biochemical Basis of the “Yang-Invigorating” Action of Chinese Yang-Tonic Herbs

**DOI:** 10.1155/2014/856273

**Published:** 2014-12-29

**Authors:** Jihang Chen, Hoi Shan Wong, Pou Kuan Leong, Hoi Yan Leung, Wing Man Chan, Kam Ming Ko

**Affiliations:** Division of Life Science, Hong Kong University of Science & Technology, Clear Water Bay, Hong Kong

## Abstract

In the practice of traditional Chinese medicine, many Yang-tonic herbs have been used for retarding the decline in bodily function and delaying the onset of age-related diseases. Our earlier studies have demonstrated that Yang-invigorating herbs/formulations protect against oxidative injury in various organs and also extend the median lifespan in mice. This lifespan extension was associated with an upregulation of cellular antioxidant status including that of mitochondria whose functional capacity is also increased by “Yang-invigorating” herbs/formulations. In this paper, we propose that triterpenes and phytosterols, which are ubiquitously found in Yang-tonic herbs, may be the chemical entities responsible for enhancing mitochondrial functional and antioxidant capacity and thus the “Yang-invigorating” action. The biochemical mechanism underlying this “Yang-invigorating” action may involve a sustained production of low levels of mitochondrial reactive oxygen species (ROS) secondary to an increased activity of the electron transport chain, with the possible involvement of mitochondrial uncoupling. The increase in mitochondrial functional capacity can retard the decline in bodily function during aging, whereas the mitochondrial ROS production is instrumental in eliciting a glutathione antioxidant response via redox-sensitive signaling pathways, which can delay the onset of age-related diseases.

## 1. The Role of Mitochondrial Decay in Aging-Related Diseases

Population aging, resulting from both longer life expectancy and declining fertility rates, has become one of the most distinctive demographic events in the 21st century in most developing and developed countries. According to the World Health Organization, between 2000 and 2050, the proportion of the world's population over 60 years of age will double from about 11 to 22% [1]. There is no doubt that aging can exert financial pressures on publicly funded healthcare systems and on long-term and income support programs in various countries.

From a biological perspective, aging is defined as an inevitable degenerative process in physiological functions and metabolic processes, ultimately leading to morbidity and mortality [2]. The incidence of neurodegenerative diseases, cardiovascular disorders, metabolic diseases, and cancers increases exponentially with age [3]. While the complex biochemical pathways leading to pathological consequences of aging remain relatively unclear, a consensus is emerging on the theory that describes a gradual decline in mitochondrial function with age, which eventually leads to progressive tissue damage resulting from oxidative stress [4–6]. The mitochondrial electron transport chain (ETC), in which numerous one-electron transfer reactions occur, has been recognized as quantitatively the most important source of reactive oxygen species (ROS) in the majority of eukaryotic cell types [7]. The mitochondrial ETC reduces oxygen molecules to water by tetravalent reduction that occurs in one-electron transfers in complex IV, with reactive oxygen intermediates being tightly bound in the complex. However, electrons leaking out from the ETC can result in the formation of partially reduced reactive oxygen species in both the mitochondrial matrix and the cytoplasm [8–10]. A large body of evidence has accumulated showing that, with advancing age, mitochondrial ROS production significantly increases in various organs, including the heart, liver, and brain, indicative of increasing oxidative stress [11–13]. However, the mitochondrial free radical theory of aging (MFRTA) has been criticized over the last decades. Inconsistent with the MFRTA, a growing number of genetic studies indicated that longevity was not affected by increasing or decreasing the level of endogenous antioxidant enzymes [14–16]. To provide a unifying view of aging and diseases, the “Double-Agent Theory” postulated that mitochondrial ROS generation produces a genetic response mimicking that triggered by infection associated increase in intracellular oxidative stress. However, the continuous mitochondrial ROS production would lead to a persistent shift in gene expression, with resultant chronic inflammation which is commonly involved in age-related diseases [17]. The abnormal mitochondrial ROS production and detoxification is accompanied by self-inflicted mitochondrial oxidative damage, which results in a significant decline in respiratory complex (I–V) activities and mitochondrial respiratory efficiency [18]. The extent of oxidative damage to key metabolic enzymes increases with age, with consequent decreases in substrate binding affinity and mitochondrial ATP generating capacity [19, 20]. Given that decay in mitochondrial structure and function is likely one of the primary causal factors in the process of aging and in the development of age-related diseases, results obtained from a large number of studies over the past two decades have suggested that the maintenance of mitochondrial functional integrity, particularly oxidative phosphorylation capacity, is important for mitigating the adverse effects of aging, as evidenced by rodent models of neurodegenerative diseases [21], heart disease [22], and aging skeletal muscle [23].

In addition, the decline in electron transport function may be directly related to the increased level of electron leakage, thereby triggering a vicious cycle of mitochondrial ROS generation [24]. To cope with an array of potentially damaging ROS, cells of aerobic organisms are believed to have developed sophisticated antioxidant mechanisms to maintain intracellular ROS at low levels within a narrow range [25]. As reduced glutathione (GSH) is the most abundant intracellular nonprotein thiol, the glutathione redox system, which involves the redox cycling of GSH and oxidized glutathione (GSSG), can be regarded as the first line of defense against ROS. The GSH/GSSG ratio has been used to assess redox status in biological systems. A reduced state of the GSH/GSSG couple appears to correlate with the proliferating state of the cell, whereas an increasingly more oxidized state is characteristic of cells during differentiation and apoptosis. The GSH/GSSG ratio decreases with aging in various organs, such as the liver, kidney, heart, and brain [26], so that mitochondrial GSH becomes more oxidized predisposing to the development of age-related diseases such as type 2 diabetes, Alzheimer's disease, and cardiovascular diseases [27]. Therefore, the maintenance of glutathione redox status is crucial for the prevention of many age-related diseases.

## 2. Yang-Invigorating Herbs and Mitochondrial Function

The practice of traditional Chinese medicine (TCM) always emphasizes the prolongation of a healthy lifespan. In this regard, many Chinese tonic herbs have long been used in the hope of retarding the age-related decline in body function and delaying the onset of age-related diseases. According to TCM theory, there are four categories of tonic herbs, namely, “Yang-invigorating”; “Yin-nourishing”; “Qi-invigorating”; and “Blood-enriching” herbs. Herbs from each category (namely, Yin, Yang, Qi, and Blood) are used for the treatment of particular types of deficiencies in body function. Holistically, the “Yang-invigorating” action involves the general upregulation of cellular activities, which is in turn critically dependent on the generation of mitochondrial ATP via the mitochondrial respiratory chain at the cellular level [28]. In this regard, some Yang-tonic herbs have been found to increase mitochondrial energy metabolism in various tissues under certain experimental conditions. For instance, treatment with* Epimedium brevicornum* increased respiratory complex IV activity and the rate of ATP synthesis in heart, brain, and skeletal muscle, thereby protecting against mitochondrial oxidative damage in aged rats [29]. Long-term treatment with an* Eucommiae ulmoides* extract produced an increase in oxidative metabolism in skeletal muscle and increased the endurance of rats to running exercise [30]. Pretreatment with a water extract of* Cynomorium songaricum* was found to restore the normal structure and function of liver mitochondria in D-galactose-challenged rats [31]. A detailed study in our laboratory has demonstrated that pretreatment with ethanol extracts of eleven Yang-tonic herbs, including* Eucommiae ulmoides*,* Cistanche deserticola*,* Cynomorium songaricum*,* Curculigo orchioides, Epimedium brevicornum*,* Dipsacus asper*,* Drynaria fortune*,* Psoralea corylifolia*,* Cuscuta australis*,* Morindae officinalis,* and* Allium tuberosum*, consistently increased mitochondrial ATP generation capacity in isolated heart mitochondria* ex vivo* and H9c2 cells* in vitro*, in association with a significant stimulatory action on the mitochondrial electron transport system [28].

In addition to upregulating mitochondrial functional capacity, Yang-tonic herbs/formulations have also been shown to enhance cellular/mitochondrial antioxidant status, thereby protecting against oxidant injury in various tissues of rats. Previous studies in our laboratory have demonstrated that pretreatment with an ethanol extract of* Cistanche deserticola* protected against myocardial ischemia/reperfusion injury* ex vivo* in rats [32], possibly through the enhancement of mitochondrial glutathione status and functional capacity [33]. Long-term treatment with a Yang-invigorating Chinese herbal formula (VI-28; composed of* Panax ginseng, Cervus nippon, Cordyceps sinensis, Salvia miltiorrhiza, Allium tuberosum, Cnidium monnieri, and Euodia rutaecarpa*) was found to increase the levels/activities of mitochondrial antioxidant components such as GSH, *α*-tocopherol (*α*-TOC), and manganese-superoxide dismutase (Mn-SOD) in various tissues, indicative of upregulation of mitochondrial redox status as a result of “Yang-invigoration,” thereby protecting against oxidative tissue damage in rat models of cerebral/myocardial ischemia-reperfusion injury, carbon tetrachloride hepatotoxicity, and gentamicin nephrotoxicity [34, 35]. More interestingly, long-term dietary supplementation with VI-28 could extend the median lifespan and mitigate age-associated declines in mitochondrial antioxidant status and functional status of various tissues in male and female C57BL/6J mice [36]. Conceivably, the retardation of decay in mitochondrial structural and functional integrity by “Yang invigoration” may have clinical applicability in delaying the onset of age-related diseases and thereby promote a healthy lifespan (i.e., “healthspan”) of humans.

## 3. Chemical Basis of “Yang-Invigorating” Action: Triterpene or Steroid-Like Compounds

**Figure 1:**
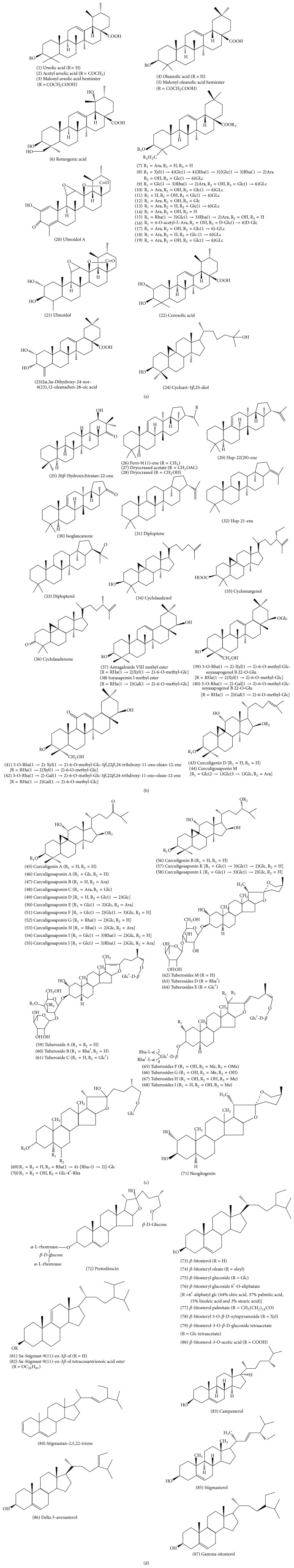
Chemical structures of triterpenes and phytosterols in Chinese Yang-tonic herbs.

Although the ability of Yang-tonic herbs to enhance functional capacity and antioxidant status of mitochondria as well as to delay the onset of aging-related diseases has been demonstrated, the chemical components responsible for the “Yang-invigorating” action of tonic herbs remain largely unknown. As a “Yang-invigorating” action involves the general upregulation of cellular activities, the enhancement of mitochondrial ATP generation can serve as a pharmacological activity marker for the activity [37]. To define the chemical basis underlying the “Yang-invigorating” action, an activity-directed fractionation of an ethanol extract of* Cynomorium songaricum* revealed that a relatively nonpolar semipurified fraction, HCY2, possessed the most potent activity in stimulating ATP generation capacity in H9c2 cells. This fraction was chemically characterized by LC-UV-MS spectrometry, and ursolic acid was found to be its major constituent. Further pharmacological studies showed that both HCY2 and ursolic acid pretreatments significantly protected against myocardial ischemia/reperfusion (I/R) injury, carbon tetrachloride hepatotoxicity, and gentamicin nephrotoxicity in both male and female rats, and this tissue protection was accompanied by an upregulation of cellular/mitochondrial antioxidant status and functional capacity [38, 39]. Another study in our laboratory using animal and cell models showed that a phytosterol-enriched fraction of* Cistanche deserticola* and its active ingredient, *β*-sitosterol, protected against hypoxia/reoxygenation-induced apoptosis in H9c2 cells and myocardial I/R injury in rats. It is likely that both of these effects were mediated by an upregulation of cellular/mitochondrial glutathione redox cycling [40]. Furthermore, asperosaponin VI, a major saponin of* Dipsacus asper *(a Yang-tonic herb), was found to protect against acute myocardial infarction in rats, an effect which might be attributed to the detoxification of lipid peroxidation products and ROS, and increases in antioxidant enzyme activities, which would result in the prevention of mitochondrial damage [41]. The aforementioned findings suggested that triterpenes or steroid-like compounds derived from Yang-tonic herbs may be the chemical components responsible for producing the “Yang-invigorating” action. The triterpenes belonging to ursane, oleanane, cycloartane, chiratane, and hopane groups and triterpene saponins as well as the steroid-like constituents in Yang-tonic herbs are summarized in (Table 1 and Figure 1).

As shown in Table 1, the ubiquitous triterpenes, like ursolic acid and oleanolic acid, and steroids, such as *β*-sitosterol and *β*-sitosteryl glucoside, were found in most of the Yang-tonic herbs. In addition, triterpene saponins were identified as major constituents in several Yang-tonic herbs. For instance, a series of triterpene saponins, which are based on the chemical structure of hederagenin and oleanolic acid, were isolated from* Dipsacus asper* [42]. Several methyl ester derivatives of olanene glycosides from seeds of* Astragalus complanatus* R.Br were reported [43]. A series of spirostanol and furostane-type oligoglycosides were also isolated from* Allium tuberosum* Rottl. ex Spreng. [44–46]. In summary, triterpenes and steroids are the major types of phytochemicals identified in Yang-tonic herbs, and this information is crucial in elucidating the biochemical mechanism(s) underlying “Yang-invigorating” activity.

## 4. Biochemical Mechanism of “Yang-Invigorating” Activity

Triterpenes and phytosterols have been reported to possess a wide spectrum of biological activities, including but not limited to antioxidant, antiatherosclerotic, antiviral, anti-inflammatory, and anticancer actions [47–51]. Although they are not usually considered as classical antioxidants, a large volume of studies have demonstrated that triterpenes such as ursane, lupane, and oleanane-types produce protective effects against oxidant injury in various organs, including heart, kidney, liver, and brain [52–54]. The tissue protection was paralleled by an improvement in antioxidant status, as manifested by increases in activities of SOD, catalase, glutathione transferases, and glutathione peroxidase [55–57]. However, the biochemical mechanism(s) underlying the antioxidant action of triterpenes and phytosterols has remained unclear.

As mentioned earlier, preliminary studies in our laboratory showed that an ursolic acid-enriched fraction derived from* Cynomorium songaricum* Rupr. and a phytosterol-enriched fraction derived from* Cistanche deserticola* Y. C. Ma invariably protected against oxidative injury, which was associated with the upregulation of cellular/mitochondrial antioxidant status as well as the functional activity of various organs [38, 39]. In these studies, both ursolic acid and *β*-sitosterol were found to increase mitochondrial electron transport chain activity. In the experiment, the electron transport in isolated mitochondria, which is driven by pyruvate and malate, was assessed by the reduction of MTT. The enhancement in mitochondrial electron transport likely contributes to the stimulatory effect of ursolic acid and *β*-sitosterol on mitochondrial ATP generating capacity, which is an indirect measure of oxidative phosphorylation [40]. A study using high throughput drug screening, whose aim was to identify disease-modifying compounds for Parkinson's disease, also showed that a series of natural triterpenes, such as ursocholanic acid and ursolic acid, significantly increased the activity of all four complexes of the mitochondrial respiratory chain and preserved intracellular ATP levels in parkin (PARK2) mutant fibroblasts [58]. In addition, a recent study demonstrated that *β*-sitosterol can become incorporated into the mitochondrial inner membrane and increase its fluidity, without affecting the fluidity of the outer mitochondrial membrane, with a consequent increase in mitochondrial membrane potential and mitochondrial ATP content [59]. The mitochondrial inner membrane fluidity was measured using fluorescence polarization probe trimethylamine-diphenylhexatriene (TMA-DPH) in that study [59]. Given that the mitochondrial inner membrane is mainly composed of unsaturated phospholipids molecules, the ability of *β*-sitosterol to increase mitochondrial inner membrane fluidity may be due to the interaction between *β*-sitosterol and the double bond of unsaturated phospholipids, which promotes membrane disorder and fluidity. In this connection, given the structural similarity between ursolic acid and *β*-sitosterol, the ability of both ursolic acid and *β*-sitosterol to stimulate mitochondrial electron transport may well be related to the increase in mitochondrial membrane fluidity.

Given that ROS are unavoidably generated during the process of oxidative phosphorylation, particularly under conditions of increased electron transport activity, the stimulation of mitochondrial electron transport by ursolic acid and *β*-sitosterol is accompanied by a small amount of mitochondrial ROS production [40, 60]. Although ROS are usually considered as culprits in the development of a number of diseases, a compelling body of evidence has demonstrated that low levels (higher than basal level but within physiological limit) of ROS also play a major physiological role in intracellular redox homeostasis and signal transduction under normal conditions [61]. The low level mitochondrial ROS-induced oxidative stress produced by prooxidant exposure, caloric restriction, hypothermia, or physical exercise elicits adaptive cellular signaling/responses that can activate various signaling pathways/transcription factors in nuclear and mitochondrial genomes, including SIRT/FOXO, electrophile response element (EpRE)/nuclear factor erythroid 2-related factor 2 (Nrf2), cAMP responsive element-binding protein (CREB), or nuclear factor (NF)-*κ*B, leading to the maintenance of homeostasis and longevity [62–65]. In addition, ROS can induce mitochondrial uncoupling that has been shown to reduce oxidative stress [66]. In our studies, we have found that the induction of mitochondrial ROS production by ursolic acid and *β*-sitosterol can elicit mitochondrial uncoupling and glutathione reductase-catalyzed glutathione redox cycling, which offer protection against oxidant injury. Ursolic acid and *β*-sitosterol not only stimulated state-3 respiration, but also caused an increase in state-4 respiration in rat mitochondria and H9c2 cardiomyocytes, with the latter being reversed by guanosine diphosphate, an uncoupling protein inhibitor. The experimental findings suggested the involvement of mitochondrial uncoupling protein activation in the “Yang-invigoration” produced by triterpene or steroid-like compounds from Yang-tonic herbs [40]. Studies by other researchers have demonstrated that both ursolic acid and oleanolic acid treatments activated the Nrf2 pathway through ROS generation, with resultant protection against cerebral ischemia and hepatotoxicity* in vivo* [54, 67]. Oleanolic acid treatment was found to dramatically increase expression of Nrf2 and its dependent-genes, such as NAD(P)H:quinone oxidoreductase 1 (Nqo1), heme oxygenase-1 (Hmox1), and glutamate-cysteine ligases (Gclc and Gclm) in rat and mouse livers [67]. Given that the activation of Nrf2 and the subsequent induction of antioxidant response genes play an important role in eliciting an adaptive response to oxidative stress [68], the ursolic acid and *β*-sitosterol-induced enhancements in glutathione redox cycling, as observed in cultured H9c2 cells [39, 40], may involve the activation of the Nrf2 pathway.

**Figure 2:**
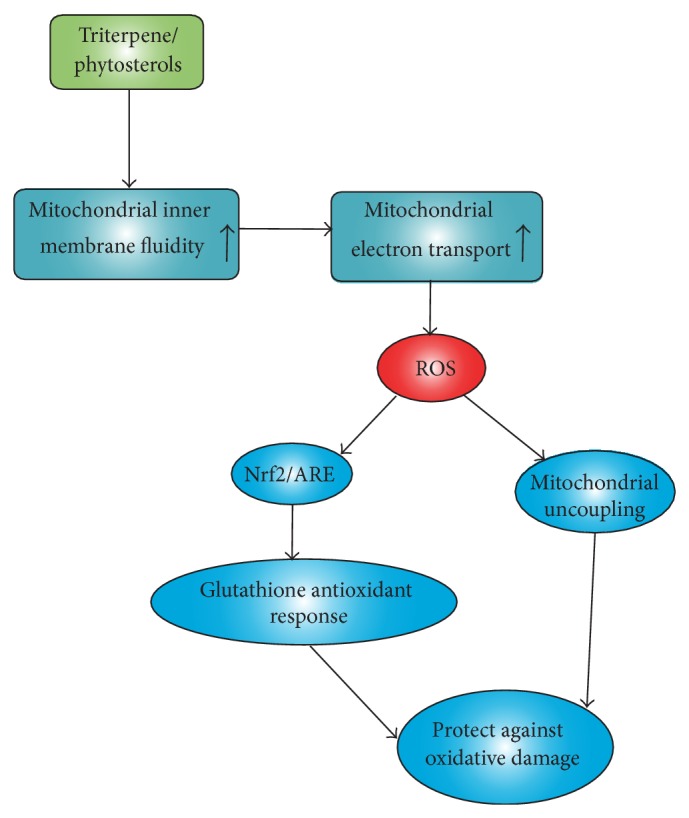
Biochemical basis of “Yang-invigorating” action.

Taken together, we therefore propose a biochemical mechanism of “Yang-invigorating” action produced by Yang-tonic herbs (Figure 2). Triterpenes or phytosterols, the active components in Yang-tonic herbs with similar chemical structure, can stimulate mitochondrial electron transport through the increase in the fluidity of the inner mitochondrial membrane, with a resultant increase in ATP generation capacity. The stimulation of electron transport is accompanied by an increase in ROS production, which triggers a retrograde response to upregulate cellular/mitochondrial antioxidant defense mechanisms through the Nrf2 pathway. In addition, mitochondrial ROS can also stimulate the activity of uncoupling protein and thereby lower the membrane potential through the dissipation of proton gradient. With a recurring “Yang-invigorating” action produced by the active components of Yang-tonic herbs, an intermittent stimulation of mitochondrial ROS production at low levels results in the prolonged activation of mitochondrial uncoupling and the upregulation of antioxidant defense components characteristic of mitohormesis, which is beneficial in retarding the decline in body function and delaying the onset of age-related diseases. To test the hypothesis, we will conduct thorough chemical analyses of biologically active extracts of Yang-tonic herbs and identify the presence of triterpenes or phytosterols. The effects of these putative active compounds on the fluidity of mitochondrial inner membrane will be investigated in association with* in vitro* and* ex vivo* bioassays on ATP generation capacity and glutathione redox cycling.

## Figures and Tables

**Table 1:** Triterpenes and phytosterols in Chinese Yang-tonic herbs.

Compounds	Herbs	Medicinal parts	Reference
Ursane-type triterpenes			
Ursolic acid (1)	*Cynomorium songaricum* Rupr.	Stem	[69]
	*Morindae officinalis* How	Root	[70]
	*Eucommiae ulmoides* Oliv.	Bark	[71]
		Leaf	[72]
Acetyl ursolic acid (2)	*Cynomorium songaricum* Rupr.	Stem	[69]
Malonyl ursolic acid hemiester (3)	*Cynomorium songaricum* Rupr.	Stem	[69]
Rotungenic acid (6)	*Morindae officinalis *How	Root	[73]
Ulmoidol A (20)	*Eucommiae ulmoides* Oliv.	Leaf	[72]
Ulmoidol (21)	*Eucommiae ulmoides* Oliv.	Leaf	[72]
Corosolic acid (22)	*Eucommiae ulmoides* Oliv.	Leaf	[72]
2α,3α-Dihydroxy-24-nor-4(23)-12-oleanadien-28-oic acid (23)	*Eucommiae ulmoides* Oliv.	Leaf	[72]
Oleanane-type triterpenes			
Oleanolic acid (4)	*Morindae officinalis *How	Root	[70]
	*Eucommiae ulmoides* Oliv.	Leaf	[72]
	*Epimedium brevicornum* Maxim.	Leaf	[74]
Malonyl oleanolic acid hemiester (5)	*Cynomorium songaricum* Rupr.	Stem	[69]
Oleanolic acid-derived triterpene saponins (7–19)	*Dipsacus asper *Wall. Ex Henry	Root	[42, 75]
Methyl ester derivatives of oleanane glycosides (37–42)	*Astragalus complanatus* R. Br	Seed	[43]
Chiratane-type triterpene			
20β-Hydroxychiratan-22-one (25)	*Drynaria fortune* J. Sm.	Root and stem	[76]
Hopane-type triterpenes			
Fern-9(11)-ene (26)	*Drynaria fortune* J. Sm	Root and stem	[76]
Dryocrassol acetate (27)	*Drynaria fortune* J. Sm	Root and stem	[76]
Dryocrassol (28)	*Drynaria fortune* J. Sm	Root and stem	[76]
Hop-22(29)-ene (29)	*Drynaria fortune* J. Sm	Root and stem	[76]
Isoglaucanone (30)	*Drynaria fortune* J. Sm	Root and stem	[76]
Diploptene (31)	*Drynaria fortune* J. Sm	Root and stem	[77]
Hop-21-ene (32)	*Drynaria fortune* J. Sm	Root and stem	[77]
Diplopterol (33)	*Drynaria fortune* J. Sm	Root and stem	[77]
Cycloartane-type triterpenes			
Cycloart-3β, 25-diol (24)	*Eucommiae ulmoides* Oliv.	Leaf	[72]
Cyclolaudenol (34)	*Drynaria fortune* J. Sm	Root and stem	[77]
Cyclomargenol (35)	*Drynaria fortune* J. Sm	Root and stem	[77]
Cyclolaudenone (36)	*Drynaria fortune* J. Sm	Root and stem	[77]
Curculigenin A, B, D (45, 56, 43)	*Curculigo orchioides* Gaertn.	Root and stem	[78]
Curculigosaponin A–J (46–55)	*Curculigo orchioides* Gaertn.	Root and stem	[78]
Curculigosaponin K, L (57, 58)	*Curculigo orchioides* Gaertn.	Root and stem	[78]
Curculigosaponin M (44)	*Curculigo orchioides* Gaertn.	Root and stem	[78]
Furostane-type saponins			
Tuberoside A–C (59–61)	*Allium tuberosum* Rottl. ex Spreng.	Seed	[44]
Tuberoside F–I (65–68)	*Allium tuberosum* Rottl. ex Spreng.	Seed	[79]
Furostane-type oligoglycosides (69, 70)	*Allium tuberosum* Rottl. ex Spreng.	Seed	[80]
Spirostane-type saponins			
Tuberoside M (62)	*Allium tuberosum* Rottl. ex Spreng.	Seed	[46]
Tuberoside D, E (63, 64)	*Allium tuberosum* Rottl. ex Spreng.	Seed	[81]
Neogitogenin (71)	*Allium tuberosum* Rottl. ex Spreng.	Seed	[82]
Protodioscin (72)	*Allium tuberosum* Rottl. ex Spreng.	Seed	[82]
Steroids			
β-Sitosterol (73)	*Cynomorium songaricum* Rupr.	Stem	[83]
	*Cistanche deserticola* Y. C. Ma	Stem	[84]
	*Morindae officinalis *How	Root	[85]
	*Drynaria fortune* J. Sm	Root and stem	[75]
	*Epimedium brevicornum* Maxim.	Leaf	[71]
	*Astragalus complanatus* R. Br	Seed	[77]
β-Sitosteryl oleate (74)	*Cynomorium songaricum* Rupr.	Stem	[74]
β-Sitosteryl glucoside (75)	*Cynomorium songaricum* Rupr.	Stem	[83]
	*Cistanche deserticola* Y. C. Ma	Stem	[84]
	*Morindae officinalis *How	Root	[86]
	*Dipsacus asper* Wall. Ex Henry	Root	[75]
	*Eucommiae ulmoides* Oliv.	Bark	[71]
	*Epimedium brevicornum* Maxim.	Leaf	[74]
	*Psoralea coryli folia* L.	Seed	[85]
	*Cuscuta australis* R. Br.	Seed	[87]
β-Sitosteryl glucoside 6′-O-aliphatate (76)	*Cynomorium songaricum* Rupr.	Stem	[83]
β-Sitosterol palmitate (77)	*Cynomorium songaricum* Rupr.	Stem	[83]
5α-Stigmast-9(11)-en-3β-ol (78)	*Cynomorium songaricum* Rupr.	Stem	[83]
5α-Stigmast-9(11)-en-3β-ol tetracosantrienoic acid ester (79)	*Cynomorium songaricum* Rupr.	Stem	[83]
β-Sitosterol-3-O-acetic acid (80)	*Cynomorium songaricum* Rupr.	Stem	[83]
5α-Stigmast-9(11)-en-3β-ol (81)	*Cynomorium songaricum* Rupr.	Stem	[83]
5α-Stigmast-9(11)-en-3β-ol tetracosantrienoic acid ester (82)	*Cynomorium songaricum* Rupr.	Stem	[83]
Campesterol (83)	*Cistanche deserticola* Y. C. Ma	Stem	[88]
	*Cuscuta australis* R. Br.	Seed	[87]
Stigmastan-2, 5, 22-triene (84)	*Cistanche deserticola* Y. C. Ma	Stem	[88]
Stigmasterol (85)	*Morindae officinalis *How	Root	[86]
	*Psoralea coryli folia* L	Seed	[85]
Delta 5-avenasterol (86)	*Cuscuta australis* R. Br.	Seed	[87]
Gamma-sitosterol (87)	*Epimedium brevicornum* Maxim.	Leaf	[74]
